# Non-Alcoholic Beverages, Old and Novel, and Their Potential Effects on Human Health, with a Focus on Hydration and Cardiometabolic Health

**DOI:** 10.3390/medicina56100490

**Published:** 2020-09-23

**Authors:** Angelos K. Sikalidis, Anita H. Kelleher, Adeline Maykish, Aleksandra S. Kristo

**Affiliations:** Department of Food Science and Nutrition, California Polytechnic State University, San Luis Obispo, CA 93407, USA; ahkelleh@calpoly.edu (A.H.K.); amaykish@calpoly.edu (A.M.); akristo@calpoly.edu (A.S.K.)

**Keywords:** non-alcoholic beverages, novel beverages, health, sparkling water, soft seltzer

## Abstract

The Beverage Guidance System has established dietary recommendations for daily intake of commonly consumed beverages including water, tea, coffee, milk, non-calorically sweetened beverages, and calorically sweetened beverages. As obesity in America continues to be a growing problem, this guidance becomes of increasing importance due to many beverages’ potential links to Type 2 Diabetes Mellitus (T2DM), Cardiovascular disease (CVD), and numerous other harmful health effects. However, the growing popularity of “better for you” beverages is causing a shift in the market, with consumers pushing for healthier beverage alternatives. Beverages simultaneously present advantages while posing concerns that need to be evaluated and considered. In this review, health effects of nonalcoholic beverages are discussed including various aspects of consumption and current trends of the beverage market such as the novel Soft Seltzer category as an alternative to Hard Seltzer and various mashups. A variety of advisory boards and agencies responsible for dietary guidelines in various countries suggest drinking water as the preferred practice for hydration.

## 1. Introduction

Between 2015–2020, beverage consumption in Americans above the age of 2 accounted for 47% of added sugars in diet, excluding milk and 100% fruit juice [1]. In 2006, the Beverage Guidance System (BGS), which divides beverages into one of six levels, was established as a means to measure, track, and decrease caloric intake through beverages. Level 1 represents the “most preferred” and level 6 the “least preferred”. This grading system was developed based on the energy and nutrient density, contribution to total energy intake and daily intake of nutrients, as well as on evidence for beneficial or adverse health effects. Typical examples of each level are as follows: (1) Water (H_2_O), (2) Tea and coffee (unsweetened), (3) Low-fat/skim milk and soy beverages, (4) Non-calorically sweetened beverages (includes “diet” beverages), (5) Caloric beverages with some nutrients and (6) Calorically sweetened beverages [2]. It is important to note that calories in non-alcoholic beverages can almost always be attributed to carbohydrate content, therefore, high sugar containing beverages typically tend to be high in calories. Beverages with >8 g/100 mL sugar are classified as high-sugar beverages [3] and are the most calorie dense.

It is recommended that of the 98 fl oz recommended daily as fluid intake from beverages, a minimum of 50 fl oz should come from water, 28 fl oz tea or coffee, 16 fl oz milk, and the remainder from alcoholic beverages, caloric beverages with some nutrients, and calorically sweetened beverages without nutrients (i.e., all the other categories combined) [2,4]. While this is only a recommended guideline, it does align with the emphasis public health and nutrition experts place on the importance of consuming low to no-calorie and nutrient dense beverages for better health and reduced caloric intake throughout the day. The beverage industry is evolving rapidly and consequently, health enhancement formulations are being introduced to create novel beverages advertised as health and hydration promotion media. Therefore, the BGS may be limited/lagging as it was established in 2006 and has not been revised to include newer beverages or changes in beverage consumption patterns. It also was never adopted by the United States Department of Agriculture (USDA), meaning there is minimal public discussion in terms of beverage consumption guidance as a part of the USDA dietary guidelines [1]. The USDA 2015–2020 dietary guidelines state that beverages account for almost 20% of total calorie intake. The recommendation is to consume primarily calorie-free beverages, especially water, and low-fat milk and 100% fruit juice, but the recommendations do not extend further. Somewhat puzzling is the USDA exemption of the 100% fruit juice from those items which need to be restricted, primarily due to their high calorie and sugar content. When beverages higher in calories are consumed, they should be accounted for and be within the recommended calorie limits [1]. Interestingly however, liquid/beverage derived calories do not seem to be as effectively “registered” by consumers as calories from food.

Only recently was the concept proposed that systematic guidance on beverage consumption may help with the development of products such as beverages lower in sugar, and dense in nutrients and phytonutrients. Furthermore, appropriate beverage choices supported by such guidance, may address existing nutrient gaps (including lower than recommended intakes of calcium in women, potassium, vitamins A, C and D from diet alone in the US), enhance phytonutrient intake, and reduce risk for chronic disease [5]. Moreover, all beverages arguably intend to produce some level of hydration, which remains a key necessary element to promote health since hydration aids maintaining temperature, lubrication and cushion of joints, supporting bowel, bladder, and kidney function as well as contributes to the protection of the spinal cord and other sensitive tissues [6]. 

Clearly, not all beverages available for consumption extend nutrition and health benefits. In this brief review we discuss health effects of different types of non-alcoholic beverages including soft drinks, coffee and tea, energy and sports drinks, and water, in an effort to systematize and consider the modern trends of innovation in the beverage industry, particularly as these relate to novel products targeting health promotion. As this review is focused on hydration and beverages consumed for hydration and/or habitual purposes, milk and juice, categorized under dairy and fruit foods groups respectively, are not discussed here. Both dairy and fruit have recommended intake levels provided by the USDA, whereas many other beverages do not. The focus of this review is to investigate the health benefits of those beverages that are not a part of these dietary guidelines for humans [1]. Literature was collected using both SCOPUS and PubMed/MEDLINE platforms with the following keywords both separately and combined: “ASB”, “nonalcoholic beverage” “carbonated water”, “sports drinks”, “energy drinks”, “caffeine”, “soft drinks”, “caloric beverages” “antioxidants”, “kombucha”, “antibacterial”, “T2DM”, “obesity”, “CVD”, “bone health”, “fracture”. Quality selection criteria included: peer-reviewed scientific publications, written in English, published after 2000-current. An initial search yielded 1053 publications. The titles and abstracts were reviewed for relevance. Of those assessed, 81 total publications were chosen based on applicability to the review and were used in addition to information from official websites/reports by Federal and International Organizations such as: Center for Disease Control (CDC), National Institutes of Health (NIH), USDA, Food and Drug Administration (FDA), World Health Organization (WHO). This review consists of a comprehensive analysis of the literature identified which focuses on nutritional components of various nonalcoholic beverages, both beneficial and detrimental for health.

In further support of the need for such an approach is the fact that while the beverage industry seems to be growing overall, traditionally strong segments of the market, including soft drinks, have begun to decline in popularity. This is primarily due to novel drinks advertising their extended health benefits [4]. This is a noteworthy trend that illustrates the consumers’ shift of where they place importance and a peaked interest for such functionality in their chosen beverage items.

## 2. Health Effects of Non-Alcoholic Beverages

### 2.1. Soft Drinks

#### 2.1.1. Calorific Soft Drinks

Soft drink (also commonly referred to as “soda”) consumption in the US seemed to have hit its peak in 2000, when 53 gallons per capita of soda were consumed. That number has declined steadily for the past 20 years. In 2018, the per capita soft-drink consumption had decreased by approximately 25% compared to that of the year 2000 (peak), reaching a mere 38.87 gallons per capita [4]. In the United States, added sugar consumption can exceed 100 g per person per day [7]. This dose is twice the maximum recommended intake (10% of a 2000 calorie diet) [8,9]. Reflective of Western trends, American sugar consumption 100 years ago is estimated at 62 g per person per day, and for 1813, the estimate is <10 g per person per day [10,11].

#### 2.1.2. Caloric Soft Drinks and Obesity

A significant body of literature strongly supports the postulation that soda consumption is associated with weight gain and development of obesity in the general population. Subsequently several studies and reviews highlight soda-type drinks as a contributor to obesity in addition to other lifestyle trends such as a sedentary lifestyle [5]. In 2009, it was reported that 62% of children in California aged 12–17 years and 41% aged 2–11 years consumed at least one soda or sweetened beverage per day. Such statistics raises significant concern from a public health perspective especially when considering that the adolescent rate of obesity is estimated to be 18% higher for individuals consuming one or more sodas a day, compared to those who do not drink soda [12]. Notably, obesity prevalence in California is estimated between 25—<30%, while in several other states in the US obesity is as high as 35% [12,13]. This, coupled with the steady increase in portion sizes recoded in the US and to a large extent globally, produces a paradigm by which larger amounts of calorific, nutrient-poor drinks are regularly consumed from an early age onwards. Between the years 1977 and 2002, calorie intake from soft drinks in the US increased by 228%, partially due to increasing portion sizes. In the 1950s, the average soda portion size was 6.5 fl oz, which conferred the equivalent of 88 kcal. In 2009, the average portion size of soda was between 12–20 fl oz, subsequently conferring 150 to 266 kcal, thus contributing to an increase of almost 200 kcal received merely from non-alcoholic drinks [14]. This energy intake, due to so-called “liquid calories,” is equivalent to 10% of a 2000 kcal daily energy intake. Moreover, when one considers the nutritional quality of those drinks typically characterized by nutrient-poor, high-sugar mixtures, it becomes evident that these items do not constitute the preferred choices for better health from an optimal healthy diet perspective. Given the rise of childhood obesity and the numerous health concerns, the World Health Organization (WHO) recommends the reduction of sugar sweetened beverages consumption in order to reduce risk for obesity and subsequent health detriments [15].

#### 2.1.3. Caloric Soft Drinks, T2DM, and CVD

Besides a higher caloric intake and the subsequent association with overweight and obesity, mounting evidence indicates additional concerns regarding higher risk of a variety of chronic metabolic diseases due to consumption of soda-type beverages, including but not limited to cardiovascular disease (CVD) and type 2 diabetes mellitus (T2DM). 

The relation between soda consumption and T2DM onset was investigated in 2037 employees in a factory in Japan [16]. Participants were evaluated over a 7-year period and reported their frequency of soda consumption, ranging from rare/never to over one serving per day, while T2DM incidence was also recorded. The crude incidence rates for T2DM of these participants who were rare soda drinkers, one serving per week, over one serving per week, one serving per day, and over one serving per day were 15.5, 12.7, 14.9, and 17.4, respectively. Furthermore, 170 participants developed T2DM. While these observations do not conclusively demonstrate a link between T2DM and soda consumption, they do indicate a potentially increased risk of T2DM onset related to such dietary practice [16].

A cohort of 116,671 female US nurses aged 24–44 comprised the Nurse’s Health Study II, a cohort study beginning in 1989. In a study conducted by Ludwig et al., these nurses participated in an investigation looking at sugar sweetened soft drinks, weight gain, and T2DM onset. This particular subset of the cohort followed 51,603 women and their dietary and lifestyle habits for eight years (1991–1999). Women with a history of diabetes or cardiovascular disease (CVD) at baseline or women who did not answer properly were excluded from the study [17]. It was found that women who drank sugar-sweetened soft drinks tended to be less active, smoke more, show an increased energy intake and decreased alcohol and protein intake. In total, 741 cases of T2DM were reported, and there was a strong association between T2DM onset and soft drink consumption. After adjusting for confounding variables, the relative risk for women consuming one or more soft drinks daily was 1.98 when compared to those who consume less than one per month. This correlation is likely due to the fact that sugar-sweetened soft drinks are calorie dense, but not nutrient dense. Increased beverage consumption can therefore lead to increased sugar intake, weight gain, and T2DM risk [17].

A 2008 study evaluated 43,960 African American women for six years (1995–2001), all of which reported not having T2DM at baseline. Out of the entire cohort, 2713 women developed T2DM during the study. In total, 17% of participants drank at least one sugar-sweetened beverage per day, and 32% drank at least one sweetened fruit drink per day. Women who consumed soft drinks and/or fruit drinks showed an increased risk for T2DM onset. Women who drank >2 soft drinks per day showed a relative risk of 1.24 when compared to those who consumed less than one per month, and those who consumed fruit drinks showed a relative risk of 1.31 when compared to those who consumed less than one per month. These numbers are particularly interesting, as African American women are twice as likely to develop T2DM than white women [18].

Individuals with T2DM are more likely to develop CVD. As sugar-sweetened soft drink consumption is closely related to both obesity and T2DM, it is likely that consumption is related to CVD risk as well. In a meta-analysis investigating CVD and sugar sweetened beverages (SSB), four studies were analyzed. This created a total of 194,664 participants, in which 7396 developed CVD. A significant association was found between SSB intake and incidence of CVD, further illustrating the adverse effects of SSB intake [19]. It is important to note that it was not specified whether SSBs were limited to soft drinks only, and this result may include other drinks such as fruit juices.

#### 2.1.4. Caloric Soft Drinks and Bone Health

The negative health effects of sugar-sweetened soft drinks extend beyond obesity, T2DM, and CVD. Heavy soft drink consumption has also been linked with hypocalcemia and osteoporosis. To better understand this connection, four groups of female rats were studied, three of which underwent bilateral ovariectomies in order to decrease levels of estrogen, known to inhibit bone resorption. Groups I and II received tap water for consumption, and III and IV received two different brands of soft drinks to consume. Bone mineral density and calcium levels were then recorded over the course of two months. It was found that rats consuming soft drinks drank three times more than those who consumed tap water, but their solid food intake was about half when compared to the tap water groups. Rats in the soft drink groups also developed hypocalcemia, and femoral mineral density was significantly decreased. To rule out these results being due to decreased solid food intake, a pair fed group was then studied. The rats consuming tap water did not develop hypocalcemia or show a decreased bone mineral density, therefore suggesting that heavy soft drink consumption is linked to reduced bone mineral density [20].

There have been some concerns expressed that carbonated soft drink consumption can increase fracture risk and lower bone mineral density, especially in children. To further investigate this, 1335 boys and girls aged either 12 or 15 years were observed for beverage consumption and fracture risk. It was found that higher consumption rates of soft drinks correlated with a decreased bone mineral density, but only in girls. This was particularly interesting as boys tended to consume more soft drinks than girls, but also consumed more milk and showed increased physical activity [21]. Another concern is that those SSBs may be displacing healthier beverages from the diet of children [21].

Similarly, 17,383 adults aged 20–75 participated in a study investigating fracture risk and soft drink consumption. Soft drink consumption was assessed in 2004, 2006, 2009, and 2011, and individuals were asked about fracture incidence and soft drink consumption patterns. It was found that after adjusting for sociodemographic and lifestyle factors and other dietary patterns, frequent soft drink consumers (at least one drink per day) had an odds ratio of 2.72 for fracture, when compared to those who did not consume soft drinks [22]. It is important to note that neither one of these studies discussed other beverage intakes, such as dairy or water intake. Therefore, it is difficult to determine whether it is the soft drink or lack of other nutrients causing the increased fracture risk. It was mentioned that this fracture risk may be due to decreased calcium and increased phosphorus intake, resulting in bone resorption [22], meaning that the combination (lack of nutrients from other beverages and poor nutrient intake in soft drinks) may be causing the fracture risk. A study investigating high soft drink consumption paired with calcium supplementation may be of use to determine cause of fracture.

#### 2.1.5. Caloric Soft Drinks and Other Negative Health Effects

Soft drink intake does not appear to provide any protective health benefits. Instead, current research aims to explore the harmful effects within these beverages that are consumed in excess by many Americans daily. There are suggestions that SSBs may largely be responsible, at least partly, for the childhood obesity epidemic in America [23,24]. The increased consumption of soda and other SSBs has paralleled the rise in obesity for decades. Specifically, it is the high-fructose corn syrup (HFCS) in soda that is often emphasized as a key contributor to the health detriment those drinks appear to be associated with. HFCS is 42–55% fructose, and the remainder is glucose. The metabolism of fructose differs from regular monosaccharide metabolism (sucrose is not included as it is not typically a part of HFCS). The physiological significance of highlighting HFCS is the increased contribution of fructose in the mixture. Fructose is of particular concern in from a metabolic and hence dietary perspective because it bypasses the first regulatory node in the glycolytic pathway, hence increasing metabolic pressure for more generation of pyruvate which in a positive energy balance scenario can be inducing fatty acid formation and thus ultimately contributing to obesity. Furthermore, as fructose needs to be converted to glucose, and this is only doable in the liver, there is a significant accumulation of glucose in the liver which again increases metabolic pressure increasing risk for ectopic lipid accumulation in the liver, thus inducing non-alcoholic fatty liver development. Fructose does not stimulate insulin release and bypasses a key-regulatory step in glycolysis so therefore provokes a unique, idiosyncratic metabolic response. Some studies have shown that fructose eventually becomes converted to glucose (in the liver), without the appropriate insulin release to match it, but further research is necessary to determine the capacity of that process. The lack of insulin response however is of particular interest in T2DM and obese individuals for whom insulin resistance and/or insufficiency is already a problem [25]. In a cross-sectional analysis, Tamez et al., reported that soda intake is positively correlated with an increase in serum C-Reactive Protein (CRP) concentration, a biomarker for inflammation and predictor of cardiovascular disease, specifically among premenopausal women. Among 825 Mexican women, a 50% higher CRP concentration was recorded among women in the highest soda intake quartile, compared with the women in the lowest quartile. Additionally, leptin was elevated among women who drink more soda, suggesting that leptin resistance may be common among patients with obesity [26]. A much larger study, looking at the risk of SSBs among Mexican women (N = 72,667) found that the median consumption of regular soda was 1.17 servings per day and per additional 1 serving incurred a 27% increase in diabetes incidence. This study confirmed an association of sugar-sweetened soda consumption and increased risk of diabetes [27].

Since insulin resistance and, in general, deregulation of glucose control are associated with increased risk for Alzheimer’s disease, there is a concern as to how SSBs may induce such risk [28]. Indeed, simple sugar intake has been associated with increased risk for cognitive diseases including Alzheimer’s disease [28,29]. Moreover, increase in simple sugars and the reduction in dietary fiber are believed to extend lasting and detrimental effects on the human gut microbiota [30].

Under daily consumption patterns, sweetened soft drinks seem to be closely linked to a series of adverse health effects, and should therefore be rarely consumed. In fact, a 10–20% decrease in consumption of these beverages could result in a 1.8–3.4% decrease in new T2DM cases and up to a 1% decrease in new CVD cases in the state of California alone. This is also projected to conserve over $620 million in savings on medical care [21], displaying the necessity and potential benefits of decreased consumption.

### 2.2. Non-Calorific Soft Drinks/Artificially-Sweetened Beverages (ASBs)

Due to growing concerns regarding the potential negative correlation of soda and obesity, consumption of artificially sweetened soft drinks has expanded as a popular alternative. The primary purpose of artificial sweeteners’ development was to substitute sugar to help consumers manage blood glucose levels better. These drinks have been typically developed so that low-to no-calories are conferred due to sweeteners, and were considered, at least initially upon their introduction, a better and healthier option as compared to the regular soft drinks [30,31,32]. However, research investigating health effects of artificially sweetened soft drinks produced data with mixed conclusions as per how they impact health especially when long-term regular consumption is considered.

#### ASBs, Obesity, and T2DM

Swithers et al., investigated the cause of weight gain in college students, frequently dubbed the “freshman 15”. In total, 172 students participated and provided a baseline blood sample within three days of arrival on campus. At the end of the school year (9 months later), samples were collected again. Weight and adiposity values were also collected. It was seen that 66 students had increased central adiposity, and there was an average of 4.0 kg weight gain. The source of these weight changes was then investigated, and it was found that erythritol, a metabolite, was significantly and positively associated with adiposity gain. While the origin cannot be pinpointed exactly, it is likely that erythritol came from increased consumption of artificial sweetener or its production during metabolism [33]. Dietary records were not collected, which would be helpful for identifying the direct source, but it does point towards a correlation between weight gain and artificial sweetener consumption. 

A multi-ethnic study published in 2009 observed diet soda consumption and its correlation to metabolic syndrome and T2DM. A total of 6814 individuals aged 45–85 self-reported demographics, lifestyle characteristics, and health history at baseline, and were then examined biannually through 2007. It was found that 14% of participants consumed >1 serving of diet soda per day, and 59% reported never consuming diet soda. Thus, 871 cases of metabolic syndrome and 413 cases of T2DM were reported throughout the study. When compared to non-consumers, those who consumed >1 serving of diet soda had a 36% greater risk of developing metabolic syndrome, and a 67% greater risk of developing T2DM, both after adjusting for confounding variables [34]. 

In the aforementioned study by Sakurai et al., in Japan, diet soda consumers were isolated from other soda drinkers. Rare soda consumers’ crude incidence rates were 1.05 while for those who consumed one serving per week was 1.70. Interestingly, there is evidence suggesting that diet soda is associated with increased risk for T2DM [16]. While not necessarily as directly intuitive, there is metabolic and biological plausibility offering a potential explanation as to how an artificial sweetener can induce risk for insulin resistance and subsequently T2DM. More specifically, the hypothesis is that artificial sweeteners’ consumption subsequently promotes overcompensation of calories via simple carbohydrates intake as it falsely activates the desire for simple sugar in the brain however negating the sweet taste satisfaction [35]. In fact, regular consumption of artificial sweeteners has been associated with decreased satiety, altered glucose homeostasis and increased caloric intake and obesity in adults [32,35]. There is also significant evidence that artificial sweeteners change the gut microbiota in a non-favorable fashion thus increasing risk for obesity, T2DM, and CVD as the microbiota extends impact on those metabolic pathologies [36].

More specifically, Suez et al., reported artificial sweeteners’ effects on glucose intolerance. In an in vivo study, the researchers added saccharin, sucralose, or aspartame, three common artificial sweeteners to drinking water of 10-week-old mice that were monitored and evaluated for changes in glucose intolerance. Interestingly, the findings revealed that all three groups receiving the various artificial sweeteners developed glucose intolerance. Furthermore, the microbiota of these mice closely resembled microbiota of humans with T2DM, suggesting those artificial sweeteners, or a preference for them, may be closely linked to T2DM onset [32]. 

It is important to note that the Suez study used saccharin only, just one of many artificial sweeteners. These results cannot be used to make assumptions on all artificial sweeteners. Sweeteners are metabolized in different ways, and some are completely degraded into amino acids, which are then absorbed into the small intestine. This means that they never reach the colon and do not interact with the gut microbiota [37]. As a result, Lobach et al., compiled a comprehensive review of artificial sweetener interactions on the gut microbiome, exploring multiple artificial sweeteners (acesulfame K, aspartame, neotame, saccharin, sucralose, and rebaudioside A). It was found that only studies involving saccharin caused changes in the microbiota. These studies tended to use levels of the sweetener well over the human Acceptable Daily Intake (ADI) [38]. For example, in a study done by Anderson and Kirkland using rats, the saccharin dose provided was 2000 times higher than the 5 mg/kg/day ADI [39]. The likelihood of saccharin significantly altering the average consumers microbiome is slim [38]. Other reviews have reported similar results, with only saccharin and sucralose showing shifts in microflora [40].

This beverage category is an area needing extensive further research. Sucralose, a prevalent non-nutritive sweetener has been documented to enhance in vitro secretion of glucagon-like peptide 1 (GLP-1), a hormone produced in the taste buds and colon. GLP-1 can cause an array of physiological effects, including delayed gastric emptying, increased satiety, suppression of glucagon secretion and insulin secretion. Brown et al. conducted a cross-over design study (N = 44) using diet soda and carbonated water to observe the effect, if any, of diet soda on GLP-1 in vivo. Participants were selected from three categories, type 1 diabetics, type 2 diabetics, or healthy (control) participants. Each participant drank either 240 mL of diet soda or carbonated water, followed by a 75 g glucose load and blood samples were collected before, at baseline and 12 more times over a 3 h period monitoring for glucose and GLP-1 levels. Results showed that diet soda increased GLP-1 secretion by 34% in healthy subjects, by 43% among type 1 diabetics and not at all among type 2 diabetics [41]. Further research is necessary to explain the absence of GLP-1 response in participants with T2DM, but it is known that when stimulated by the presence of glucose, GLP-1 secretion causes the release of insulin, suppression of glucose and therefore lowers serum blood glucose [41]. Such findings call into question the current suggestion by many physicians and other health professionals to switch to diet soda when counseling patients with T2DM. 

The Academy of Nutrition and Dietetics’ (AND) position on artificial sweeteners is that they can be enjoyed when “consumed within an eating plan that is guided by current federal nutrition recommendations, such as the Dietary Guidelines for Americans and the Dietary Reference Intakes, as well as individual health goals and personal preference” [42]. The American Diabetes Association (ADA) position is that artificial sweeteners may be a good alternative to SSBs although water is considered still the best option [43]. However, a recent advisory from the American Heart Association (AHA) states that: “Nonetheless, there is a dearth of evidence on the potential adverse effects of low-calorie sweeteners (LCS) beverages relative to potential benefits. On the basis of the available evidence, the writing group concluded that, at this time, it is prudent to advise against prolonged consumption of LCS beverages by children” [44]. As there is apparently a notable lack of properly designed randomized controlled long-term studies to assess efficacy of artificial sweeteners in different populations, whereas observational studies often remain confounded due to reverse causality and often yield opposite findings [45], it remains unclear how safe those compounds really are, especially in long-term exposure. 

Concerns regarding artificially sweetened beverages (ASBs) include “alterations of the composition of intestinal bacteria, reconditioning of the brain when faced with ASBs that are 200 times sweeter than sugar, and hypoglycemia caused by discordant insulin secretion when a sweet taste is present without a corresponding increase in serum glucose” [46]. Despite these concerns, Big Soda and others in favor of diet soda consumption present evidence to the contrary. For example, two studies reported that sucralose had no effect on the fasting plasma glucose, HbA1c value or fasting serum C-peptide levels. It was concluded that sucralose had no effect on glucose homeostasis in individuals with T2DM and there is no evidence of reason to discourage its consumption on the basis of discordant insulin [46,47]. This area of nutrition is still largely unexplored and needs considerable further research before a definitive conclusion can be reached.

### 2.3. Caffeinated Beverages

#### 2.3.1. Coffee and Tea

Coffee and tea are an integral part of many Americans’ day to day routines. In fact, in 2015, consumers spent $74.2 billion on coffee in the United States alone [48]. Tea is the second most consumed beverage worldwide, second only to water [49]. There is a general perception that both beverages have numerous health benefits, though evidence-based established effects may not fully support the general public’s perception. Several of the studies reviewed focused on coffee and tea consumption together, and therefore tea and coffee are not separated into individual groups. 

#### 2.3.2. Health Benefits of Coffee and Tea

Matcha green tea has been rumored to boost brain function and increase longevity. In order to investigate this concept, for a study evaluating the effects of matcha on antioxidant status, mice were grouped and fed one of 7 different experimental diets for 4 weeks: control, high fat, high fat with 0.0025%, 0.05% or 0.075% matcha supplemented. Serum total cholesterol and triglyceride levels of the high fat diet with 0.05% matcha were significantly lower than the mice on the high fat diet alone. Moreover, low density lipoprotein (LDL) and blood glucose levels were decreased as well in this group. These findings argue in favor of a cardioprotective effect of matcha tea possibly due to its natural antioxidant and bioactive compounds [50].

A study initiated in 1994 examined 40,530 Japanese adults aged between 40–79 years without history of stroke, coronary heart disease, or cancer. Participants were followed for 11 years. Of the cohort, 4209 participants died during the 11-year follow-up period of whom, 892 due to CVD and 1134 due to cancer. Consumption of green tea, a typical beverage in the Japanese tradition, was inversely associated with total mortality and CVD, with this relationship being stronger in women. However, there was no evidence that green tea consumption was inversely associated with mortality due to cancer in either sex [51].

A 2009 study investigated coffee, decaffeinated coffee, and tea consumption and its relation to T2DM onset. A total of 457,922 participants reported on their coffee and tea consumption and T2DM status was considered. Data were collected either through 24 h dietary recalls or self-reported food frequency questionnaires. An inverse relationship between all three beverages and T2DM risk was reported, and for every additional cup of coffee consumed, the risk decreased by 7% [52]. Notably, these results come from unsweetened drinks. It is believed that the suggested benefits are not necessarily extended by caffeine, but it was reported that benefits may be attributed more likely to other factors such as magnesium, lignans, and chlorogenic acids. The direct function of these compounds is not clear, while numerous studies support that that these nutrients and phytochemicals are involved in glucose homeostasis and insulin secretion [52,53,54]. 

In another study focusing specifically on coffee, 88,259 US women aged 26–46 years and no history of T2DM at baseline were investigated. The women reported a regular coffee consumption throughout the course of the 10-year follow-up period. At completion, 1263 women developed T2DM. However, after adjusting for confounding factors, evidence of decreased T2DM onset for regular coffee drinkers was clear. In fact, relative risk of T2DM for those who drank one cup a day was 0.87 when compared to non-drinkers and was as low as 0.53 for those who drank 4 cups a day. It was also found that the type of coffee (decaffeinated, instant, or caffeinated) made no difference in terms of T2DM risk, thus the type of coffee was not a risk modifier in this study. These results argue that benefits from coffee consumption relative to T2DM risk are caffeine independent, thus arguably caffeine is not seemingly attenuating risk for T2DM [55].

#### 2.3.3. Potential Negative Effects of Coffee and Tea

While coffee appears to have numerous health benefits, its consumption may also present certain risks, one such being an elevated risk of lung cancer. Five prospective studies and 8 case-controlled studies comprised a meta-analysis investigating coffee intake and lung cancer. Combined, it was found that there was a significant positive association between coffee intake and lung cancer, specifically at highest coffee intake. An increase in coffee consumption of 2 cups/day was associated with a 14% increase in risk of lung cancer. While it is difficult to state causality due to potential confounding variables that may or may not have been considered, there does appear to be some correlation between coffee consumption and development of lung cancer [56]. 

Increased coffee consumption has also been associated with elevated serum cholesterol levels. In 7213 women and 7368 men aged 20–54 years that reported coffee consumption, high-density-lipoprotein (HDL) cholesterol, and triglyceride levels were tested. It was found that coffee consumption was positively associated with total cholesterol levels and triglycerides in both men and women, and remained significant after adjusting for age, smoking, and alcohol consumption [57].

### 2.4. Energy Drinks

Energy drinks initially entered the market in the United States in 1997 and have experienced increased demand for more products since their inception. In fact, in 1997, one brand dominated the market and by the year 2006, 500 new brands were commercially available worldwide. Regulation of these drinks has always been complicated, causing contradictory health claims [58]. 

#### 2.4.1. Potential Benefits of Energy Drinks 

Energy drinks sometimes contain ginseng, taurine, or natural products high in caffeine such as guarana. The safety and properties of energy drinks products rich in these compounds was investigated in an extensive literature review. It was found that caffeine levels tend to fall between 80–300 mg of caffeine per 8 oz serving, and these drinks can have as much as 35 g of sugar as well. These high levels were found in four popular energy drinks. Authors further reported no negative effects found when using ginseng, guarana, or taurine. Four cases of caffeine-related deaths were identified, as well as four separate cases of seizures due to energy drink consumption. However, it does appear that neither guarana, ginseng, nor taurine present a risk in terms of negative health effects at reasonable dosing [59]. It does remain an issue however what a reasonable and acceptable upper limit might be for those compounds, especially when considering long-term regular consumption. 

Due to the content of energy drinks in taurine, caffeine and B vitamins, it has been debated that they may be effective for increased mood and performance. In total, 36 volunteers, all moderate caffeine users, participated in 3 different studies. Heart rate, alertness, choice reaction time, and blood pressure were recorded at baseline. The participants then consumed either carbonated mineral water or energy drink (study 1), energy drink or no drink (study 2) and still water or energy drink (study 3). In study 1, it was found that carbonated mineral water resulted in decreased blood pressure and heart rate when compared to energy drink consumption, but subjective mood was more greatly affected by energy drinks. In study 2, reaction time was significantly improved with energy drink consumption when compared to no drink. Alertness and aerobic endurance were increased as well. In study 3, memory performance was significantly better in those that consumed energy drink, opposed to still water. Therefore, in this study it appears that energy drink consumption greatly contributed to improved brain function as related to memory. However, all participants in this study were students at the University of West England, age 18–30 years, hence these results can only be discussed in regard to a younger overall healthy population [60]. 

#### 2.4.2. Negative Health Effects of Energy Drinks

While the aforementioned caffeinated beverages are associated with health benefits, caffeine specifically presents numerous health risks for certain groups, including children and adolescents, and pregnant and lactating women. For children and adolescents, high caffeine levels found in energy drinks can have numerous negative effects. In a review of energy drink consumption in children and adolescents, it was found that 30% to 50% of the population sample investigated consume energy drinks. This fact raises concerns as it was also reported that these drinks could cause seizures, cardiac abnormalities, and other health issues in this particular group. In fact, of the 5448 caffeine overdoses in the US in 2007, 46% were under 19 years. Due to the lack of regulation and understanding surrounding these drinks, children and adolescents are placed at increased risk and reasonable discretion should be exercised relative to consumption [61]. Major health organizations like the American Academy of Pediatrics suggest that children under the age of 12 years should not eat or drink caffeine-containing foods or drinks [62]. A recent review of the evidence has concluded that in children, caffeine doses >400 mg can cause physiological, psychological, and behavioral harm, in particular in subgroups of children, such as those with psychiatric or cardiac conditions [63].

Pregnant women are currently recommended to consume no more than 200 mg/day of caffeine, or approximately one daily 12 oz cup of coffee due to risk of decreased birth weight according to the American College of Obstetricians and Gynecologists (ACOG) [64]. To determine caffeine consumptions effect on birth weight, 1011 women were surveyed within 3 days of delivery in a Yugoslavian hospital. Birth weight, sex, presence of congenital malformation, and gestational age at delivery were recorded. In total, 13.5% of women reported consuming no caffeine during pregnancy. The remainder consumed an average of 133.44 mg/day. After adjusting for confounding variables, it was found that birth weight decreased as caffeine consumption increased. For mothers who consumed >141 mg, birth weight was reduced by 114 g, a reduction determined to be significant [65]. 

A second study investigating the effects of caffeine on birth weight focused solely on intake during the third trimester. Caffeine intake of 111 mothers of small-for-gestational-age (SGA) infants were compared to intake of 747 mothers of non-SGA infants. Caffeine intake from coffee, tea, soft drinks, and chocolate was estimated. It was found that mothers with SGA infants showed increased caffeine intake during their third trimester when compared to mothers of non-SGA infants, with the mean consumption being 281 mg/day, while this risk was highest for male infants. No increased risk was reported for female infants [66].

According to the most recent committee opinion from the ACOG, reaffirmed in 2020, with regards to miscarriage or preterm birth, moderate caffeine consumption (less than 200 mg per day) does not appear to be a major contributing factor. Additionally, the relation between caffeine with intrauterine growth restriction, or between high caffeine intake and miscarriage, have yet to be determined [64]. Notably, mounting evidence from both epidemiological and animal studies suggests several harmful effects of maternal gestational caffeine exposure, even from doses previously considered “safe”, including adverse cardiometabolic effects in the offspring and subsequent generations [67].

Energy drinks and caffeine have mixed health effects, and particularly at-risk groups should be aware of amount consumed, especially in long term. While studies indicate that coffee and tea are both effective at preventing T2DM onset, elevated caffeine levels appear to have a particular effect on children. Seeing as data surrounding energy drinks is less clear, adolescents should take particular care when consuming these beverages, and should limit intake significantly. According to the Committee on Nutrition and the Council on Sports Medicine and Fitness from the American Academy of Pediatrics, rigorous review and analysis of the literature has revealed that caffeine as well as other stimulant substances found in energy drinks should not be part of child/adolescent [68].

### 2.5. Sports Drinks

Another category of popular non-alcoholic beverages are sports drinks, marketed at improving recovery and performance for high-level athletes. Their effectiveness has long been under scrutiny, since there is argumentation supporting that the benefits do not practically extend beyond hydration and recovery [69].

In a comprehensive review assessing 60 studies, data relating to the effectiveness of sports drinks on varying athletes was collected. Exercise duration was separated into short (less than one hour), prolonged (1–4 h), and ultra-endurance (more than 4 h) and whether the exercise was continuous or intermittent (all exercises were cardio based). Focus was on drinks containing <10% carbohydrate, as these are recommended for consumption before and after exercise, and not for carbohydrate loading. Glycogen-compromised individuals benefitted, regardless of exercise duration, from drink consumption, but evidence for glycogen-sufficient individuals was less clear. It was concluded that consumption of sports drinks during intermittent and prolonged exercise, and as well as before prolonged exercise, all appeared to improve performance [69].

In a separate study, recovery was investigated in 44 high school football players using a 7% glucose polymer beverage with electrolytes or a non-nutrient non-electrolyte placebo. Mean and peak sprint velocities, body weight, plasma volume, glucose levels and insulin were evaluated in these players. Researchers concluded that the pre- to post-scrimmage differences in velocity and body weight were similar between both groups, but in the placebo group insulin and glucose levels were much higher. Plasma volume was significantly lower in the placebo group as well. It was concluded that carbohydrate drinks are effective at maintaining plasma volume during recovery [70].

While sport drinks necessity continues to be debated, it does appear that they do make an impact on recovery, especially in terms of glucose levels. In another study evaluating the effects of commercially available sports drinks on substrate metabolism and subsequent endurance performance in a postprandial state, the carbohydrate sports beverage with additional protein was shown to maintain insulin production during endurance cycling at 70% $\dot{V}$O_2_max in the postprandial state [71]. This is an interesting finding because if we consider intake of such a beverage in a non-exercise regime then the induction of insulin secretion may plausibly over time contribute to insulin resistance. Additionally, one needs to consider the amount of simple carbohydrate typically found in those drinks (which is considerable) and how that can contribute to significant simple sugar intake raising significant concerns associated with simple sugar intake discussed earlier [71].

### 2.6. Kombucha-Type Drinks

Kombuchas origin is not quite clear, reportedly anywhere between 200–2000 years old. Its rise in popularity only began in the US in the 1980s, due to beliefs that the compounds could increase T-cell counts and support the compromised immune systems of individuals living with HIV/AIDS [72]. What started more as a grassroots movement later exploded into an industry boom for reasons attributed to possible health benefits, the rise in craft brewing, or the increased interest in fermentation as a whole. Kombucha can be alcoholic, but for the purposes of this review, we are discussing the non-alcoholic or low level (<0.5%) brews only) [72].

#### 2.6.1. Antimicrobial Properties of Kombucha

Kombucha has been discussed for its supposedly antimicrobial activities, with limited evidence in support. In a 2000 study, the antimicrobial study of kombucha during fermentation was investigated. Values of pH and acetic acid levels were monitored throughout the 4-day fermentation period, given acetic acid antimicrobial properties. Staphylococcus aureus, Shigella sonnei, Escherichia coli, Campylobacter jejuni, Salomnella enteridis, Salmonella typhimurium, and eight other bacterial strains were all shown to be sensitive to kombucha. However, even at neutral pH and thermal denaturation, Escherichia coli, Shigella sonnei, Salmonella typhimurium, Salmonella enteritidis, and Campylobacter jejuni were still sensitive, suggesting that the antimicrobial properties observed should be attributed to additional factors beyond mere acetic acid [73].

In another study investigating antimicrobial properties and antifungal properties, both green and black teas were used. Kombucha was made traditionally, in this case only black tea was used, and fermented for 21 days. Both acidic and neutral kombucha samples were tested. Both forms displayed antimicrobial properties, regardless if they were gram negative or positive, the strongest of which being *S. epidermidis*, *M. luteus*, *L. monocytogenes*, and *P. aeruginosa*. Green tea was found to have stronger antimicrobial properties, as the acidified form was inhibitory for all bacteria. Black tea was only effective against *L. monocytogenes* and *P. aeruginosa*. In terms of antifungal properties, all tested yeast other than *C. krusei* were sensitive to kombucha treatment. There was more variation among green and black tea blends in terms of which was more effective, as black tea tended to be active against more yeast strains. Therefore, depending on where the interest in health lies, it may be more beneficial to choose a green or black tea blend. Also, seeing as neutral samples also displayed antifungal and antimicrobial properties, it is evident again that other ingredients different that acetic acid responsible for these properties [74].

#### 2.6.2. Antioxidant Activity in Kombucha

Kombucha is also believed to have increased antioxidant activity, attributed to the polyphenols commonly found in kombucha [75]. Higher polyphenol contents have been linked to increased antioxidant activity [76]. Antioxidants are believed to help decrease low-density lipoprotein (LDL) cholesterol [77]. However, it is important to note that kombucha is only believed to have antioxidant activity, and any claims have not yet been approved by food safety authorities.

To determine the effect of kombucha on a high-cholesterol diet, mice were fed either standard chow or a high cholesterol diet (either a diet enriched in cholesterol, lard oil, or cholate) for 12 weeks. The diet was then supplemented with traditional kombucha tea, D-saccharic acid-1,4-lactone (DSL, black tea), modified kombucha (a tea brewed with a single Gluconacetobacter sp. responsible for high levels of DSL), or no supplement. Body weight, food intake, and antioxidant status was recorded throughout the 12 weeks. It was found that black tea, traditional kombucha, and modified kombucha all functioned as antioxidants to free radicals and effectively inhibited LDL oxidation, while modified and traditional kombucha exhibited a stronger effect compared to DSL. The modified kombucha tea showed the highest antioxidant activity, and it was therefore concluded that brewing kombucha from a single strain may be more beneficial [77]. 

Seeing as kombucha has numerous health benefits, it may be worth investigating as a dietary supplement to receive beneficial probiotics to positively impact the gut microbiome and increase antioxidant activity. This may also be effective at decreasing T2DM risk as T2DM onset has been linked to an unhealthy microbiome [20] and oxidation. Kombucha is also low in calories, averaging around 30 calories/8 fl oz, suggesting that intake will likely not lead to significantly increased calorie consumption [78].

### 2.7. Sparkling Water-Based Beverages

Due to notable negative health effects of soda and similar soft drinks, consumer demand has been high and sustained for alternative tastes and products addressing multiple consumer needs including improved dietary intake. One such product is sparkling water, which has experienced a popularity boom in the past 10 years. In fact, in 2009, 400 million liters of sparking water was sold in the United States. By 2019, that number almost doubled, reaching just under 800 million liters sold, while projections predict a continued increase in demand for sparkling water and/or sparkling water-based beverages [79].

#### 2.7.1. Hydration Capabilities of Sparking Water

It appears that sparkling water may have more to offer than simply the “it tastes good” and “it has no negative health effects” often mentioned by consumers. While not thoroughly studied, it is believed to hydrate as well, if not better than, water, considering the better electrolyte levels and may be notably effective in decreasing intestinal distress [80]. To investigate this concept, Rosario et al. evaluated the effects of carbonated water on patients with dyspepsia and secondary constipation. More specifically, 21 patients were randomly assigned to two groups- tap water or carbonated water and consumed the respective water exclusively for 15 consecutive days. It was found that dyspepsia was significantly reduced in the carbonated water group when compared to the control (tap-water), and constipation was reduced as well. Satiety scores were also recorded and were found significantly reduced in the carbonated water group. The authors thus concluded that carbonated water may be effective at reducing dyspepsia and constipation and may decrease hunger as well [80].

Similarly, 19 healthy women participated in a study investigating carbonated water on appetite sensation. Women were assigned water (tap-water), carbonated water, or no beverage to consume after an overnight fast. Gastric motility and fullness levels were then recorded after consumption. It was found that carbonated water resulted in increased fullness scores and an improved satiating effect [81]. Weight loss was not recorded, but carbonated water may be useful at appetite suppression and thus possibly support weight loss efforts [81].

Eight male volunteers participated in an intermittent cycle exercise experimental study to explore hydration effects of carbonated water. Within 30 min of exercise completion, they consumed one of the following four drinks: (A) a glucose solution, (B) a sodium chloride drink, (C) a potassium chloride drink, (D) a solution of glucose, sodium chloride, and potassium chloride. Both potassium chloride and sodium chloride are commonly found in sparkling water. Individuals then underwent electrolyte analysis to determine rehydration effects of the beverages. It was found that ingestion of beverage A resulted in higher urine output and a greater net negative fluid balance the following day in comparison to all other beverages, hinting at the idea that glucose is not effective at rehydration, and may be functionally diuretic. Beverages B and D resulted in the lowest net negative sodium balance the following day, and negative potassium balance was greater after consumption of beverage A and B. Therefore, it is evident that those beverages that contained electrolytes (i.e., B, C, D) appeared to hydrate better than beverages that did not [82]. While this is a small sample size, it does suggest that carbonated water can potentially offer better hydration compared to tap water. 

#### 2.7.2. Potential Negatives of Carbonated Water Consumption

While it seems that carbonated water hydrates as well as water and may be beneficial for various reasons, some sensitive groups may wish to limit their intake. Individuals experiencing overactive bladder are recommended to decrease carbonated beverage intake. A total of 6424 women over 40 years old participated in a survey analyzing urinary symptoms and their connection to carbonated beverage intake (including water), as well as tea, coffee, wine, beer and fruit juice. It was observed that women who drank as little as one carbonated beverage per week had an elevated risk of stress incontinence (SI), and those who consumed a carbonated beverage daily had an almost 2X higher risk of SI than those who consumed daily. Consumption of carbonated beverages also increased risk of overactive bladder onset, although risk was not as high as that for SI [83]. Therefore, while carbonated beverages may be beneficial for hydration and by extension overall health, they may negatively affect certain individuals who should monitor consumption to minimize potential symptoms. Another consideration with carbonated water as an alternative to tap water is in relation to fluoridation, especially for young children. Given that tap water is typically fluoridated in the US it may be beneficial for better support of dentition in early ages when children are still being trained in terms of oral hygiene and optimal habits [84] while carbonated water is not fluoridated. 

The combination of novelty and health in new beverage proposals may be in line with the needs and desires of the modern food scene and consumer demand [85]. Sparkling water-based beverages with natural added ingredients are emerging as novel drinks with significant consumer traction as they are claiming a niche at the intersection of safe, healthy, and enjoyable drinks. Moreover, when such sparkling water-based drinks are infused with vitamins, natural antioxidants, and/or other natural bioactive compounds that have established benefits, those constitute good options for health-conscious consumers interested in novel drinks with functionality. Other options include the addition of amino acids for support of special groups such as athletes or elderly at risk for sarcopenia [86]. It has been proposed that appropriate beverages can potentially address existing micronutrient gaps in the population, enhance phytonutrient intake, and reduce the risk for chronic disease [5]. Thus, consumers are able to receive the advantages of sparkling water with additional possible health benefits without the concerns of “diet” products with artificial constituents and preservatives. There are interesting recent efforts such as the H2O/H_2_♡ novel Soft Seltzer, non-alcoholic seltzer flavored with dealcoholized wine, from Sonoma, California [87]. This constitutes a promising example of such a modern approach [87], as an alternative to hard seltzers [88] containing alcohol such as White Claw introduced in 2016 [89]. Other examples of novel beverages include the mashups category whereby coffee or non-alcoholic wine are carbonated.

In Table 1, we present a summary of the studies reviewed on health effects of nonalcoholic beverages (Table 1).

### 2.8. Importance of Hydration in the Context of CVD and T2DM

There is significant evidence supporting the importance of hydration in terms of metabolic health. More specifically, hypohydration is shown to increase the risk for CVD and T2DM development via a variety of potential mechanisms. Mechanistically, angiotensin II is the principal hormone of body fluid regulation and when activated by a state of hypohydration stimulates thirst, an appetite for sodium, the release of ADH/vasopressin and vasoconstriction in small arterioles to increase total peripheral resistance [90,91]. Chronic elevation of angiotensin II often promotes inflammation and therefore can serve as an indicator for other chronic human diseases; hence state of dehydration may be directly connected to metabolic dysfunction. As cells gradually increase their state of dehydration, metabolism of free fatty acids and amino acids to pyruvate or acetyl-coA becomes compromised and a growing dependence on glucose as the main fuel source is observed, a condition that is shown to promote the development of obesity [90]. Interestingly, angiotensin II was established as the driving factor triggering the series of events that follow when total fluid intake is insufficient leading to a metabolic switch compromising health, the use of angiotensin converting enzyme (ACE) inhibitors for conditions like high blood pressure, obesity and chronic kidney disease became rather popular as a medication for many noncommunicable diseases. The question for future researchers, however, is as to whether ACE inhibitors solve the problem or rather act as merely a temporary control of symptoms.

In addition to elevated release of angiotensin II, suboptimal fluid intake or hypohydration fosters an increased circulation of vasopressin, also known as antidiuretic hormone. The surrogate marker for vasopressin, copeptin, is more commonly evaluated in the field due to its longer half-life. Elevated circulating copeptin is associated with a host of comorbidities including enhanced risk of metabolic syndrome with abdominal obesity, type 2 diabetes mellitus, hypertension, coronary artery disease, heart failure, cognitive impairment, microalbuminuria, chronic kidney disease, inflammatory bowel disease, cancer and premature mortality [92,93,94,95]. The effects of increased water intake on plasma copeptin were examined in 82 healthy adults. Participants who originally consumed low-to-moderate total fluid intake were placed into an intervention that significantly increased plain water intake. Over a 6-week period that closely followed and monitored participants, a 24.7% decrease in circulating copeptin levels was recorded, from 5.18 to 3.90 pmol/L [92]. Additionally, individuals drinking less than 1.2 L of water per day were found to have significantly high levels of plasma vasopressin, and therefore copeptin, when compared to their counterparts who consumed 2 liters per day [93]. 

Vasopressin was shown to stimulate the release of glucocorticoids leading to upregulation of serum glucocorticoid-regulated kinase-1 (SGK1) [95]. SGK1 stimulates Na^+^/K^+^-ATPase, carriers and ion channels, participates in the regulation of transcription factors as well as many cellular functions including the organization of the cytoskeleton, cell volume regulation, cell survival, cell proliferation and hormone release. Additionally, SGK1 stimulates appetite for high salt-containing foods, influencing greater intake and creates the potential for a predisposition of hypertension, renal failure, tumor growth, diabetes, cardiac failure and obesity. Low water drinkers have a more active glucocorticoid cascade systems and higher plasma cortisol levels [95]. Therefore, the cascade effects of hypohydration, including but not limited to the release of SGK1 and stress hormones, creates predisposition for largely preventable chronic diseases, the leading cause of mortality and morbidity in the US and elsewhere. 

Water may be considered an under-researched nutrient, thus future research is necessary to investigate the effects of different categories of beverages on water balance, the mechanisms and the factors involved, such as the role of copeptin in the pathogenesis of T2DM, obesity, and CVD. Notwithstanding, the available evidence collectively suggests a marked potential for a great public health interest: the protective benefits of increased water intake. Nutrition recommendations and guidelines in the US by American Cancer Society (ACS) [96], American Heart Association (AHA) [97], Center for Disease Control (CDC) [98] Dietary Guidelines for Americans 2015–2020 [99], Canada’s Dietary Guidelines [100], Australian Dietary Guidelines [101] and Food-Based Dietary Guidelines in Europe (European Food Safety Agency) all propose and/or recommend water as the best choice for hydration [102], while the WHO recommend moderation of SSB and not regular consumption [103]. Finally, notably not all safe drinking water is equally optimal due to variations in its quality such as composition of minerals, both qualitatively and quantitatively [104].

## 3. Conclusions

While there are currently no federally regulated guidelines for beverage consumption in the United States, the BGS works well as a baseline. Water is the ideal choice for replacing physiological water losses, though it is unrealistic to ask individuals to only consume water, and in doing so, numerous potential health benefits of various beverages would be overlooked. Given the less than optimal dietary intake of micronutrients in individuals and population groups related to a potential mineral suboptimal status, along with a variation in mineral content of tap water or bottled water depending on location, certain individuals and populations may require alternative hydration sources in order to optimize water and mineral balance, and the related physiological consequences. Thus, other beverages beyond tap and bottled water, can be beneficial for health. An updated, federally regulated outline for beverages is necessary in the United States and beyond and is currently missing. The beverage market continues to grow and evolve, just as dietary patterns do, and guidelines should reflect an interest in these changes. While SSBs may taste favorably from a consumer’s standpoint, there are significant concerns as per weight gain, and health specifically regarding T2DM and CVD risk, as well as cognition and bone fragility. This is not limited to calorically sweetened beverages only, as artificial sweeteners have also been linked to negative health outcomes. With the increasing introduction and interest in novel beverages such as kombucha, sparkling water, and Soft Seltzer, options for nonalcoholic beverages outside of water and soda continue to grow. Moderate consumption of these novel beverages may support improved hydration and health and have the potential to decrease soft drink consumption potentially functioning as an alternative and a tool for addressing health issues related to cardiometabolic disease. 

## Figures and Tables

**Table 1:** Summary of studies on non-alcoholic beverages with potential benefits/concerns regarding health.

Beverage Category	Recommended Daily Intake * (fl oz)	Potential Benefit (s)	Concern (s)	References
Caloric Soft Drinks	0–8	None	Excess calorie consumption leading to obesity, T2DM risk, CVD risk, decreased bone density	1, 10–27
Noncaloric Soft Drinks	0–16	Fewer calories	Decreased satiety, increased calorie uptake, T2DM risk	1, 30–47
Coffee and Tea	0–40	Low to no calorie, decreased T2DM risk, lower cholesterol and triglyceride levels (tea)	Lung cancer risk (coffee), elevated cholesterol levels (coffee), decreased birth weight	1, 48–57
Energy Drinks	0–8	Increased brain function, memory, reaction time	Elevated heart rate, increased blood pressure, excess consumption may lead to caffeine related deaths or seizures	1, 59–68
Sports Drinks	0–16	Improved performance, plasma maintenance, beneficial for glycogen deficient individuals	Caloric, not always necessary	1, 69–71
Kombucha	0–16	Antimicrobial and antifungal properties, increased antioxidant activity, low calorie	Caloric-excess consumption could lead to weight gain	1, 72–78
Sparkling Water	20–50	Hydrates as well as water, better electrolyte levels, decreased intestinal distress, increased fullness, higher satiety levels	Increased risk of overactive bladder, stress incontinence	1, 79–89

* Recommended daily intake is not as per American Society of Nutrition guidelines but rather as per researchers used doses; T2DM: Type 2 Diabetes Mellitus; CVD: Cardiovascular disease.
